# Bilateral Chorea-Ballism Associated With Non-ketotic Hyperglycemia: A Case Report

**DOI:** 10.7759/cureus.63221

**Published:** 2024-06-26

**Authors:** Ouafae Abbassi, Amina Ali Kako, Yassine Mebrouk

**Affiliations:** 1 Department of Neurology, Centre Hospitalier Universitaire (CHU) Mohammed VI, Oujda, MAR; 2 Department of Pharmacy, Mohammed First University, Oujda, MAR; 3 Department of Neurology, Mohammed Vl University Hospital, Oujda, MAR

**Keywords:** magnetic resonance imaging, diabetes mellitus, caudate and lenticular nuclei, chorea-ballism, non-ketotic hyperglycemia

## Abstract

The intricate workings of uncontrolled diabetes and its effects on the nervous system are not fully understood. However, it is known that this condition can lead to various neurological manifestations, including altered consciousness and epileptic seizures. In this case study, a 66-year-old woman presented with abnormal ballistic movements and chorea due to severe hyperglycemia. The results of her brain MRI suggested diabetic striatopathy. Fortunately, her symptoms improved significantly with the normalization of blood glucose levels and appropriate medical intervention. While the exact pathophysiology remains elusive, there is speculation that dysfunction of dopaminergic activity may play a role. The management of these complications involves stabilizing blood glucose levels and providing symptomatic relief. Despite the limited understanding of the underlying mechanisms, these neurological complications generally have a positive prognosis.

## Introduction

Chorea and ballism are neurological syndromes characterized by involuntary muscle contractions, with ballism being more severe and characterized by larger, proximal movements compared to the finer, distal movements seen in chorea [1]. While these conditions can have various causes, including vascular, autoimmune, drug-induced, metabolic, and infectious factors, one rare but significant etiology is severe non-ketotic hyperglycemia in poorly controlled elderly diabetic patients [1]. The impact of diabetes on the neurovascular system extends beyond peripheral neuropathy, often presenting complex challenges in diagnosis and management for physicians [2]. In particular, neurological manifestations such as hemiballismus can arise, representing a spectrum of choreiform movements associated with uncontrolled diabetes [2]. This case highlights the importance of recognizing and addressing neurological complications in diabetic patients, underscoring the need for comprehensive care and management strategies.

## Case presentation

A 66-year-old woman with vascular risk factors, including hypertension on monotherapy and non-insulin-dependent diabetes on oral antidiabetics (metformin 2 g per day), was admitted to the emergency department for a treated ketoacidosis decompensation and was declared discharged. Since then, the patient has presented with abnormal movements and has been readmitted. These involuntary, repetitive, and generalized movements involve all four limbs and the face. These movements led to a significant deterioration in her ability to perform essential daily tasks, including cooking, dressing, and managing household chores. The abnormal movements only disappeared completely during deep sleep. Apart from the involuntary, repetitive, and generalized movements (Video 1), other neurological and general examinations were unremarkable. There was no family history of neurological disorders.

**Video 1:** Generalized chorea

Physical examination revealed abnormal movements of the generalized chorea-ballismus type, more severe on the left side. The finger-nose maneuver showed right-sided dysmetria, which was difficult to assess given the severity of the movements. All cranial nerves were evaluated and found to be normal. No motor or sensory deficits to light, touch, pain, position sense, or vibration were noted. All deep tendon reflexes were normal, and there were no deficits in muscle tone or strength (5/5 in all four extremities).

**Figure 1:**
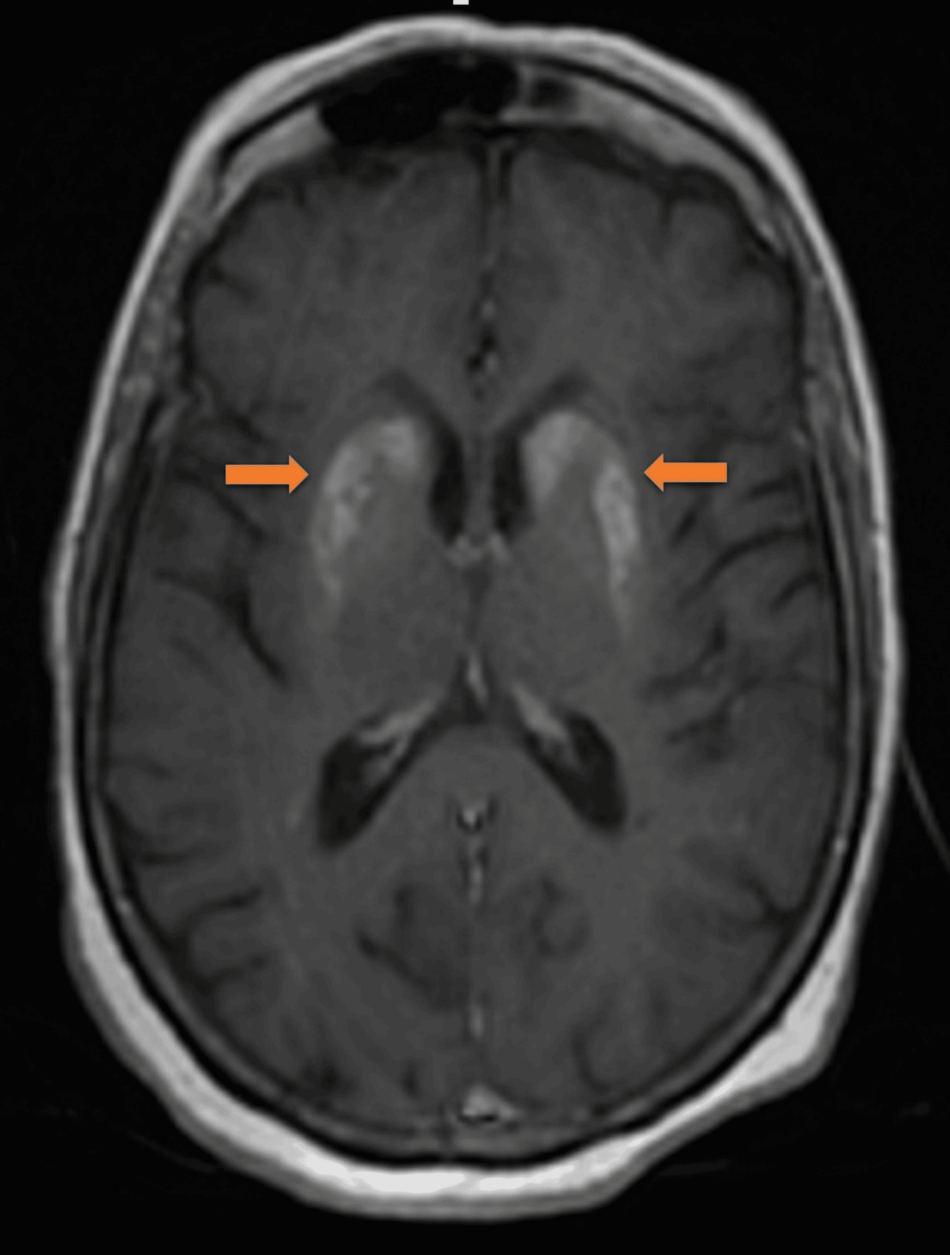
Axial MRI T1 revealing hyperintensities in the striatal region bilaterally MRI: magnetic resonance imaging

**Figure 2:**
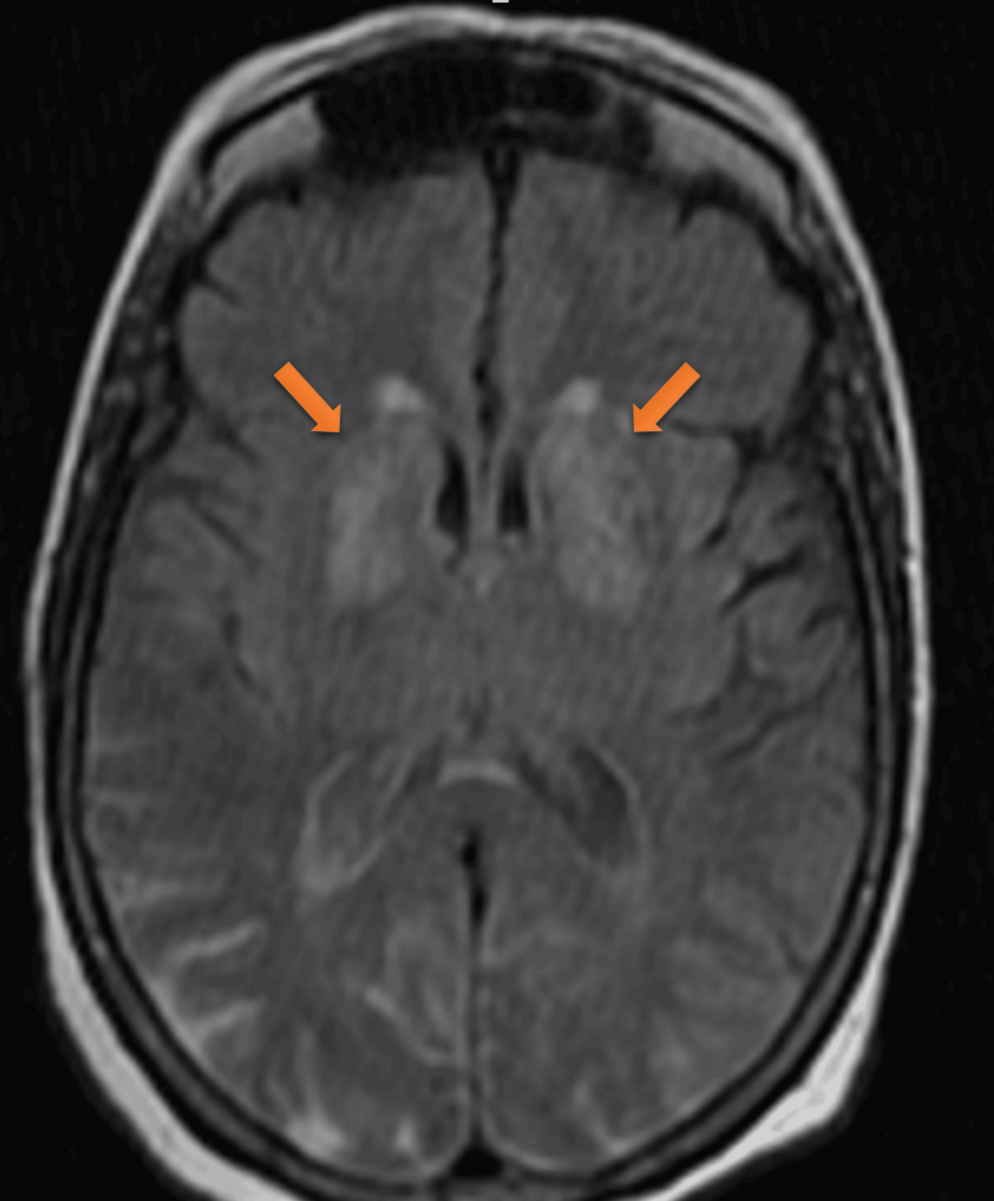
Axial MRI T2 revealing hyperintensities in the striatal region bilaterally MRI: magnetic resonance imaging

Examination of vital signs revealed nothing significant. The initial laboratory work-up revealed hyperglycemia at 2.25 g/l (normal range 0.70-1.10 g/l), creatine at 17 mg/l (normal range 7-14 mg/l), an estimated glomerular filtration rate at 31 ml/mn/1.73 m² (normal range 95 ml/mn (+/-20 ml/mn)), and hemoglobin A1C at 7.8% (normal range 4.1- 5.7%). Serology for the hepatitis virus, HIV, and syphilis was negative. Hematological and endocrine assessments, including a complete blood count with differentials and thyroid-stimulating hormones, were normal. MRI on admission revealed a T1 and T2 hypersignal involving both the caudate and lenticular nuclei without diffusion restriction (Figures 1-2). The hypothesis of diabetic striatopathy was evoked first, given the clinical context.

Following a period of hospitalization, the patient's blood sugar levels returned to within the normal range. However, there was minimal improvement in the range of motion observed in the patient's left arm. A 2 mg/ml haloperidol solution (1.5 mg - 1.5 mg - 2 mg) was therefore administered orally daily, resulting in a slight improvement after one week. A decision was made to increase the doses (2 mg - 2 mg - 2.5 mg ), and a week later, the patient was seen to have experienced a near-complete regression of movements. The haloperidol was continued at the previously administered dosage. Two months later, the patient exhibited no evidence of abnormal movements. Haloperidol was discontinued.

## Discussion

These movements were first reported as a complication of hyperglycemia without ketosis by Bedwell in 1960 [3]. Stroke is the leading cause of acquired chorea-ballismus, followed by nonketotic hyperglycemia as the second most prevalent cause [4,5]. Other causes of hemiballism include head trauma, amyotrophic lateral sclerosis, neoplasms, tuberculomas, demyelinating plaques, the human immunodeficiency virus, metabolic disturbances (sodium, magnesium, calcium, hyperthyroidism), and drug use (anticonvulsants, levodopa, oral contraceptives, and neuroleptics).

The pathophysiology of the onset of abnormal movements during hyperglycemia without ketosis remains poorly understood, although several hypotheses have been proposed. The brain structures involved, including the basal ganglia, essentially the subthalamic nucleus, the pallido-subthalamic pathway, the striatum, and, to a lesser degree, the cerebral cortex and thalamus, are susceptible to cellular energy depletion during hyperglycemia [6]. This condition can result in the inhibition of the tricarboxylic acid cycle, which in turn leads to the alternative use of GABA as an energy source [7]. The prevalence of these atypical movements in postmenopausal women may be attributed to hormonal alterations that influence dopamine functionality [8]. Hypertension and diabetes in elderly subjects can also contribute to choreic movements by causing cerebrovascular lesions [9].

Our patient had long-standing poor metabolic control, with an HbA1c of 7.8% and nonketotic hyperglycemia of 2.25 g/dL. Although the disease is usually unilateral, in less than 10% of cases, the basal ganglia are affected bilaterally [4,10], as was the case in our patient. We thoroughly investigated potential autoimmune, infectious, and toxic triggers, all of which were ruled out. Furthermore, there was no familial precedent for symptoms resembling chorea.

The use of MRI as a modality to confirm diagnosis is undoubtedly the best. Hyperglycemia without ketosis is associated with the presence of spontaneous hypersignals in the basal ganglia, as observed in previous studies [11,12]. The neuropathological nature of these alterations remains a matter of contention. These lesions are thought to be related to hemorrhagic lesions, myelinolysis, post-anoxic calcifications [13], or hemosiderin deposits following petechial suffusions [8]. Some authors have proposed that these alterations are associated with the presence of gliosis, which is characterized by the presence of abundant astrocytes, as demonstrated during a biopsy of a hyperintense putamen or during an autopsy [14].

Therapeutic management is primarily based on strict glycemic control, which has been shown to result in an improvement in neurological symptoms, even in the absence of neuroleptics or with minimal doses. The symptoms may disappear over a period of weeks or months. With regard to imaging, it is possible that abnormalities may persist for longer periods, with resolution occurring days or weeks after normalization of blood sugar levels. Anticholinergic drugs, such as deutetrabenazine and tetrabenazine, may be indicated and may result in clinical improvement, particularly if symptoms persist following the correction of hyperglycemia. Haloperidol has also been employed in the treatment of this condition, although if the patient does not respond, clonazepam, tetrabenazine, and tiapride may be considered as alternative options [15]. The use of haloperidol is contingent upon a number of variables, including advanced age and comorbidities, which may elevate the risk of short-term mortality and influence the selection of the most efficacious treatment. Furthermore, surgical procedures such as thalamotomy [16] and deep brain stimulation may be considered in rare cases of refractory disease [17].

## Conclusions

Abnormal movements, such as ballismus and chorea, are uncommon complications of hyperglycemia. However, they are generally associated with a favorable prognosis. Although the correlation between clinical symptoms and anatomical alterations is well established, the precise pathophysiology remains undetermined. It is of paramount importance to gain a deeper understanding of this association, as it has significant implications for therapeutic approaches.
